# Intradermal melanocytic nevus with lymphatic nevus cell embolus: A case report

**DOI:** 10.3892/ol.2013.1704

**Published:** 2013-11-25

**Authors:** HYUN-SOO KIM, SANG HWA LEE, HYUNG-SIK MOON, YOUN WHA KIM

**Affiliations:** 1 Department of Experimental Analysis, Aerospace Medical Center, Republic of Korea Air Force, Cheongju, Chungcheongbuk-do 363-849, Republic of Korea; 2 Department of Pathology, Aerospace Medical Center, Republic of Korea Air Force, Cheongju, Chungcheongbuk-do 363-849, Republic of Korea; 3 Department of Dermatology, Aerospace Medical Center, Republic of Korea Air Force, Cheongju, Chungcheongbuk-do 363-849, Republic of Korea; 4 Department of Pathology, School of Medicine, Kyung Hee University, Seoul 130-701, Republic of Korea

**Keywords:** intradermal melanocytic nevus, benign metastasis, lymphatic nevus cell embolus, mechanical transport theory

## Abstract

The current study presents a rare case of intradermal melanocytic nevus with lymphatic nevus cell embolus. A 26-year-old male presented with a slowly enlarging, pigmented nodule on the back, measuring 1 cm in diameter. Histological observations of the lesion were typical of an intradermal melanocytic nevus. The most notable feature of this nevus, however, was an aggregate of nevus cells within a lymphatic vessel of the upper dermis. The nevus cells observed within the lymphatic lumen demonstrated characteristic morphological features of type A nevus cells. The cells were round-to-cuboidal, exhibited abundant cytoplasm with well-defined cell borders and formed nests. In addition, the nevus cell aggregate was lined by flattened endothelial cells. Nevus cell aggregates occur in the collagenous framework of lymph nodes, however, the mechanism by which nevus cells are deposited in lymph nodes has been a source of interest and controversy. The histological observation presented may be regarded as support for the mechanical transport or benign metastasis theories, which posit transfer of nevus cell emboli, via lymphatics, from a cutaneous nevus to the draining regional lymph node. Due to its rarity, a lymphatic nevus cell embolus creates diagnostic and management issues for pathologists and clinicians. This observation must not be interpreted as evidence of malignancy, but must be assessed in context with the associated histological features of the lesion.

## Introduction

Intradermal melanocytic nevi are common, benign, pigmented skin tumors formed by proliferation of dermal melanocytes. A number of notable, uncommon changes may be observed in intradermal melanocytic nevi. In particular, their association with lymphatic invasion is an extremely rare phenomenon. To date, only two cases of an intradermal melanocytic nevus with lymphatic invasion have been described in the literature (1,2).

The observation of nevus cell aggregates in the lymph nodes has been of practical and academic significance (3), and various hypotheses have been presented to explain their origin. The current study presents a case in which an aggregate of nevus cells was observed within a lymphatic vessel of the upper dermis. The observation of a lymphatic nevus cell embolus supports the hypothesis that the nevus cells are likely to be transferred, via lymphatics, from a cutaneous nevus to the draining lymph node.

## Case report

A 26-year-old male presented to the Department of Dermatology (Aerospace Medical Center, Republic of Korea Air Force, Cheongju, Korea) with a slowly enlarging nodule on the back. The patient had no personal or family history of malignant melanoma. A physical examination revealed a brown-colored, pea-sized cutaneous nodule on the back. A clinical diagnosis of melanocytic nevus was determined and the lesion was excised with adjacent skin. For light microscopic examination, the tissue was immediately preserved in 10% buffered formalin. Following 48 h of formalin fixation, the specimen was embedded in paraffin, routinely processed and stained with hematoxylin and eosin.

**Figure 1:**
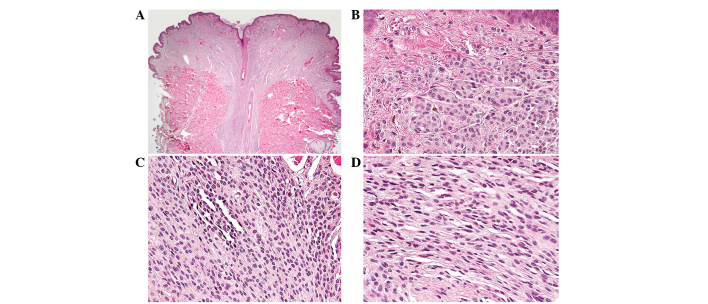
(A) Scanning view of the resected specimen. The lesion measured 1 cm in diameter and 0.3 cm in height (hematoxylin and eosin; magnification, ×12.5). Lesional cells in the upper, middle and lower dermis presented characteristic morphological features of types (B) A, (C) B and (D) C nevus cells, respectively (hematoxylin and eosin; magnification, ×200).

**Figure 2:**
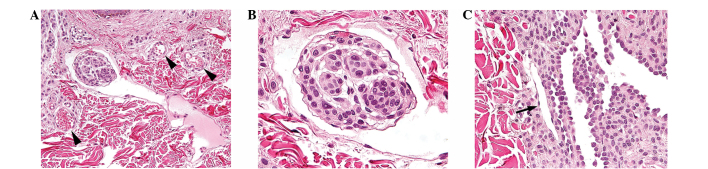
(A) An aggregate of nevus cells was observed within a lymphatic vessel of the upper dermis. In contrast to adjacent small blood vessels with red blood cells and small, round lumina, indicated by the black arrowheads, the lymphatic vessel contained proteinaceous fluid without red blood cells and showed a large lumen (hematoxylin and eosin; magnification, ×100). (B) The lymphatic nevus cell embolus demonstrated characteristic morphological features of type A nevus cells; a round to cuboidal shape, well-defined cell borders, abundant cytoplasm and nest formation. In addition, it was lined by flattened endothelial cells, identical to the endothelial cells lining the lymphatic lumen (hematoxylin and eosin; magnification, ×400). (C) A clear distinction in morphology was identified between a true lymphatic vessel, indicated by the black arrow and the pseudovascular spaces formed by nevus cells, indicated in the right half of the image. The lumina of the pseudovascular spaces anastomosed irregularly with each other and were lined by nevus cells with cuboidal contours and round-to-oval nuclei, and not by the vascular endothelial cells with flattened nuclei and greatly attenuated cytoplasm. Moreover, the nevus cells formed intraluminal papillary projections and specific floating individual nevus cells were also present (hematoxylin and eosin; magnification, ×200).

Grossly, the resected specimen consisted of elliptical skin with a pigmented nodule measuring 1.0 cm in diameter and 0.3 cm in height. Fig. 1A illustrates a scanning view of the lesion. Histologically, the lesional cells in the upper, middle and lower dermis presented characteristic morphological features of types A, B and C nevus cells, respectively. The type A nevus cells (Fig. 1B) in the upper dermis were round-to-cuboidal, showed voluminous cytoplasm containing variable amounts of melanin granules and formed nests. Normal epidermis overlying a nest of nevus cells was identified and junctional activity was absent. The type B nevus cells (Fig. 1C) in the mid-dermis, which were distinctly smaller than the type A nevus cells, were arranged in well-defined aggregates or cords and contained less cytoplasm and melanin. The type C nevus cells (Fig. 1D) in the lower dermis were elongated and possessed spindle-shaped nuclei. The cells were arranged in strands and rarely contained melanin. The decrease in cell size and melanization and the progression from nests to cords to more neuroidal spindle cells with dermal descent, often referred to as maturation, were observed. No mitotic figures were identified. These observations were typical of an intradermal melanocytic nevus; the most notable feature of this nevus, however, was an aggregate of nevus cells within a lymphatic vessel of the upper dermis (Fig. 2A). The large lumen of this lymphatic vessel contained proteinaceous fluid without red blood cells. By contrast, adjacent small blood vessels contained red blood cells and exhibited round, narrow lumina. The nevus cells observed within the lymphatic lumen demonstrated characteristic morphological features of type A nevus cells. The cells were round-to-cuboidal, showed well-defined cell borders and abundant cytoplasm and formed nests. In addition, the nevus cell aggregate was lined by flattened endothelial cells, identical to the endothelial cells lining the lymphatic lumen (Fig. 2B). Based on these observations, this aggregate of nevus cells was considered to be a lymphatic nevus cell embolus. No evidence of dysplastic change or malignancy was observed. Serial sections for additional slides were performed to confirm the lymphatic origin of the endothelial cells using immunohistochemistry, but the nevus cell aggregate was no longer detectable. The patient provided written informed consent.

## Discussion

Prior to the diagnosis of a lymphatic nevus cell embolus in the present study, the possibility of pseudovascular space being misidentified as a lymphatic vessel was considered. Melanocytic nevi are commonly associated with clefts or slits of nests, resembling lymphatic or vascular spaces. These pseudovascular spaces have been mainly identified as artifacts of tissue processing and may simulate the lymphatic invasion of malignant melanoma. In the present case, the vascular channel exhibiting a nevus cell embolus contained no red blood cells, showed a large lumen and was lined by a single layer of flattened endothelial cells, indicating a true lymphatic vessel. In addition, Fig. 2C (obtained from the slide that exhibited a lymphatic nevus cell embolus) clearly demonstrates the morphological differences between a true lymphatic vessel and pseudovascular space formed by nevus cells. The lumina of the latter showed intraluminal papillary projections and a few floating nevus cells. The lumina anastomosed irregularly with each other and were lined by nevus cells with cuboidal contours and round-to-oval nuclei, and not by vascular endothelial cells with flattened nuclei and greatly attenuated cytoplasm. These observations clearly identified the space as a true lymphatic vessel and excluded the possibility of a pseudovascular space.

Since the first description in 1931 (3), there have been a number of cases of benign nevus cells within lymph nodes reported in the literature (3–8). However, the histogenesis of this lesion remains unclear. The prevailing theories concerning the mechanism by which nevus cells become incorporated into lymph nodes include arrested migration of neural crest progenitor cells during embryonic development (5,8) and the transfer of nevus cell emboli, via lymphatics, from a cutaneous nevus to the draining regional lymph node, also termed mechanical transport or benign metastasis (8,9).

The following are among the observations that favored arrested migration (10): a) The concurrence of embryonic migration of melanocytic precursors and development of the lymphatic system (11); b) the presence of a blue nevus in non-cutaneous sites, such as the uterine cervix, vagina, prostate, spermatic cord and seminal vesicles (2,8); c) the usual capsular location with sparing of the sinuses; d) the rarity of observing metastatic melanoma and nevus cells in the same lymph node (9); and e) the common observation of congenital nevi in association with nodal nevus cell aggregates, indicating that anomalous migration is responsible for the nodal and cutaneous observations (11). Moreover, the morphological pattern of the distribution of nevus cells in nests and strands in the collagenous capsule and trabeculae of lymph nodes is distinct from the occupation of the subcapsular sinus observed in metastatic disease.

However, certain aspects of the association provide equally compelling support for the mechanical transport theory, including the following arguments (10): a) there are examples of intranodal deposits of other tissues, such as endometrium, endosalpinx and breast epithelium; b) nevus cells are not found in lymph nodes draining non-cutaneous sites (9); c) non-cutaneous nevus cell aggregates are found only in association with lymph nodes (12); d) melanocytes arrested in their migration through the dermis are bipolar, whereas, in the majority of cases, nevus cells in lymph nodes have the oval or cuboidal contours encountered in conventional cutaneous melanocytic nevi (13); and e) nevus cell clusters are found within cutaneous lymphatics and in afferent lymphatics of lymph nodes (1,2,11,14), which are not known to have a role in the embryonic migration of neural crest-derived cells. Consistent with the observations of two previous cases (1,2), an endosalpinx aggregate of benign nevus cells in association with intradermal melanocytic nevus was observed in the present case report. The histological findings may be regarded as support for the mechanical transport theory.

While the observation made in the current study contributes a piece to the puzzle of nodal nevi, it does not yield definitive information concerning the mechanism underlying this phenomenon. Considerable research is required prior to being able to fully understand the process of nodal involvement by nevus cells. However, it is clear that such cases require investigation with great care, and considerable caution must be exercised in interpreting the results. Moreover, it is essential that pathologists educate surgeons and oncologists with regard to the occurrence of nevus cell aggregates in lymphatics and lymph nodes.

In conclusion, the current report presents a unique case of an intralymphatic nevus cell aggregate in association with an intradermal melanocytic nevus. To the best of our knowledge, the current report presents the third case of a lymphatic nevus cell embolus observed in an intradermal melanocytic nevus. The histological observation presented may be regarded as support for the mechanical transport theory, which posits lymphatic transfer of nevus cell emboli from a cutaneous nevus to the draining regional lymph node. This observation must not be misinterpreted as evidence of malignancy, but must be assessed in context with the associated histological features of the lesion.
